# Diverse community leaders’ perspectives about quality primary healthcare and healthcare measurement: Qualitative community-based participatory research

**DOI:** 10.1186/s12939-021-01558-4

**Published:** 2021-10-18

**Authors:** Kathleen A. Culhane-Pera, Shannon L. Pergament, Maiyia Y. Kasouaher, Andrew M. Pattock, Naima Dhore, Cindy N. Kaigama, Marcela Alison, Michael Scandrett, Mai See Thao, David J. Satin

**Affiliations:** 1 Minnesota Community Care, Inc., 895 E 7th, St. Saint Paul, MN 55106 USA; 2 Program in Health Disparities Research, University of Minnesota, 717 Delaware St. SE, Minneapolis, MN 55414 USA; 3 Department of Family and Community Medicine, University of Minnesota, 420 Delaware Street SE, Minneapolis, MN 55455 USA; 4 Minnesota Health Care Safety Net Coalition, 1113 East Franklin Ave #202B, Minneapolis, MN 55404 USA; 5 Department of Anthropology, Global Religions and Cultures, University of Wisconsin-Oshkosh, 800 Algoma Blvd, Oshkosh, WI 54901 USA

**Keywords:** Primary care quality metrics, Healthcare inequities, Pay-for-performance, Value-based payments, Community-based participatory research (CBPR)

## Abstract

**Background:**

Healthcare quality measurements in the United States illustrate disparities by racial/ethnic group, socio-economic class, and geographic location. Redressing healthcare inequities, including measurement of and reimbursement for healthcare quality, requires partnering with communities historically excluded from decision-making. Quality healthcare is measured according to insurers, professional organizations and government agencies, with little input from diverse communities. This community-based participatory research study aimed to amplify the voices of community leaders from seven diverse urban communities in Minneapolis-Saint Paul Minnesota, view quality healthcare and financial reimbursement based on quality metric scores.

**Methods:**

A Community Engagement Team consisting of one community member from each of seven urban communities —Black/African American, Lesbian-Gay-Bisexual-Transgender-Queer-Two Spirit, Hmong, Latino/a/x, Native American, Somali, and White—and two community-based researchers conducted listening sessions with 20 community leaders about quality primary healthcare. Transcripts were inductively analyzed and major themes were identified.

**Results:**

Listening sessions produced three major themes, with recommended actions for primary care clinics.

#1: Quality Clinics Utilize Structures and Processes that Support Healthcare Equity.

#2: Quality Clinics Offer Effective Relationships, Education, and Health Promotion.

#3: Funding Based on Current Quality Measures Perpetuates Health Inequities.

**Conclusion:**

Community leaders identified ideal characteristics of quality primary healthcare, most of which are not currently measured. They expressed concern that linking clinic payment with quality metrics without considering social and structural determinants of health perpetuates social injustice in healthcare.

## Background

Healthcare quality measurements in the United States illustrate disparities in healthcare quality when examined by racial/ethnic group, socio-economic class, insurance, sex/gender identity, and geographic location [1]. The contributing factors, one of which is the creation of the measurement themselves, are legion. Healthcare quality measurements historically were created by insurers, professional organizations, and government agencies to measure aspects of care they considered important [1]. Incorporating the voices of diverse communities would help understand the needs of the whole population rather than just those in privileged positions. Communities experiencing social injustices caused by historical, racial, and systemic violence will only continue to be placed in harm’s way [2] with implementation of quality metrics designed by others unless their voices are heard, and their needs are addressed. Recent work has engaged more diverse communities in describing barriers to accessing care and maintaining health [3]. Yet, relatively little research has examined quality measurement in these communities. Several qualitative studies have examined providers’ perspectives on quality metrics and impact on patients [4–6], including those primary care providers (PCPs) working across the socioeconomic spectrum in both safety-net clinics and higher resource clinics [7]. To our knowledge, no studies have sought diverse community perspectives on defining and measuring quality primary care.

Reimbursement through pay-for-performance, value-based payment, and accountable care organizations all rely upon relevant and appropriate quality metrics. Pay-for-performance alone is a significant modifier of healthcare resources internationally, with published literature in at least 14 Organisation of Economic Cooperation and Development (OECD) countries and 35 additional low and middle-income nations [8, 9]. Differences between healthcare quality scores for providers serving high or low socioeconomic status populations across multiple practice settings are well-documented [10–12]. Linking payment to quality metrics exacerbates healthcare disparities [13–15]. This is a result of focusing exclusively on specific clinical markers rather than including patient-defined metrics of quality such as access and patient-centeredness [12]. In addition, social and structural determinants of health (SDOH), which are particularly relevant to the diverse communities in our study, are rarely considered in quality measurement [16]. No studies, to our knowledge, have examined diverse community members’ definitions of quality healthcare, perspectives about how quality care measurements could affect healthcare disparities, and responses to connecting payment for primary care with quality metrics.

Our study aimed to amplify the voices to those most impacted by, yet absent from, these conversations. Through community-based participatory research (CBPR) [17], we explored the viewpoints of diverse urban community leaders in the Minneapolis-Saint Paul MN metropolitan area, asking, “What constitutes quality primary healthcare?” Our specific research questions were: How do diverse community members define, perceive and experience quality primary healthcare? What recommendations and priorities do they have to improve primary healthcare? How do they respond to financial reimbursement of primary care clinics based on their quality scores?

The analysis and discussion of our results applies the healthcare quality and equity theoretic lens shared by Starfield [18], and later Stange and Etz [19]. Like Starfield, we ascribe to the International Society for Equity in Health’s definition of equity as the absence of systematic and potentially remediable differences in one or more aspects of health between groups of people characterized socially, geographically, or demographically [20]. The perspectives of the community leaders in this study can, and should, inform the process going forward to enhance systems of quality measurement to ward health equity for all.

## Methods

### Setting

The Minneapolis-St Paul metropolitan area in Minnesota USA has over 3.6 million people, with 75.1% Whites, 8.6% African Americans, 6.6% Asians, 6% Hispanics, and 0.5% Native Americans; and with 10.9% foreign born, including 28% from Africa (mostly from Somalia) [21]. The healthcare quality in Minnesota has been highly ranked nationally [1] although the disparities between Whites and minority communities are high [22], particularly when analyzed by geography [23]. In 2017, the Minnesota legislature directed state agencies to assess and make recommendations for an improved state mandated healthcare quality metric system. In response, the Minnesota Health Care Safety Net Coalition, a coalition of healthcare organizations in the metropolitan area with expertise in clinical care and quality measurement, formed the Quality Measurement Enhancement Project (QMEP) to conduct two research projects about healthcare quality measurement in order to bring clinician and patient voices into the Minnesota state process. The QMEP research project about clinicians’ perspectives has been published [7]. This QMEP research project involved obtaining perspectives of community leaders from diverse communities.

### Design

In order to effectively engage with community members as partners in the research process, the QMEP team utilized a community-based participatory research (CBPR) process [17]. We convened and trained a Community Engagement Team (CET) consisting of one community researcher as a CET Community Lead from each of seven communities: Black/African American, Hmong, Latino/a/x, Lesbian-Gay-Bisexual-Transgender-Queer-Two Spirit (LGBTQTS), Native American, Somali, and White. We chose these communities because they are from the most populous communities in the metropolitan area. With input from the QMEP team, the CET team chose the research aims, designed and conducted the qualitative methods, and completed a participatory qualitative thematic analysis. The qualitative methods included 4-h Listening Sessions with community leaders that began with an overview presentation about healthcare quality measures in Minnesota, followed by 90-min community-specific breakout focus group discussions led by each group’s respective CET Community Lead, and ended with an interactive large group discussion that was the first step in participatory analysis.

To recruit participants to the Listening Sessions*,* each CET Community Lead from the seven communities identified three to five community leaders from their respective communities. The inclusion criteria were individuals between the ages of 18–85 years, who were recognized as or were renowned as community leaders from the seven groups in the Twin Cities metropolitan area. The seven CET Community Leads, utilizing their extensive community networks to identify community leaders who would be broadly knowledgeable about the challenges their communities face in seeking and accessing health and mental health care, contacted and invited five Black/African American, five LGBTQTS, four Hmong, five Latino/a/x, five Somali, four Native American, and two White community leaders to participate. Of these identified 30 people, 20 people agreed to participate. Prior to joining, we reviewed the consent information sheet with participants; the study was determined exempt by the University of Minnesota Institutional Review Board.

We held two Listening Sessions and one phone interview to accommodate participants’ schedules; community leaders from the Black/African American and Native American communities attended one session and the leaders from the other groups attended the other session. After the healthcare quality presentation, each CET Community Team Lead led a focus group with their community in the group’s preferred language (English, Hmong, Somali or Spanish). The focus group questions elicited experiences and perspectives about what their community wanted in quality primary healthcare. (See Table 1 for small group questions). Reconvening in a large group, each small group reported their three to five major discussion points and community leaders engaged in an initial high level participatory analysis discussion [24] comparing and contrasting these key discussion points across participating communities in order to identify the themes that were most important to them. The small group sessions were audiotaped and note takers from each community captured discussions in both the small and large group sessions. The one telephone interview was audiotaped and notes were taken.

**Table 1:** Small Group Questions for Listening Sessions

1. **Community’s health**	
*Please think broadly about your community.*	
1.1 How do people in your community define health?	
1.2 What are the most important things that positively or negatively affect the health of your community?	
**2. Quality clinical health care for people from your community**	
*Please think about optimal health care in primary care clinics, for people in* your *community in MN. What makes for positive (good quality) patient experiences at primary care clinics?*	
1.1 What aspects of relationships with clinic staff do people most want from good medical care?	
2.2 What aspects of clinic process do people most want from good medical care?	
2.3 What health results do people most want from good medical care?	
**3. Recommendations for quality clinical health care**	
3.1 Prioritize your identified qualities. Please share your top 5 issues with the group.	
3.2 How do you feel about Minnesota state aligning financial payment with these prioritized issues?	
3.3 Consider the context of people’s lives, known as “social determinants of health (SDOH)”.	
3.3.1 How would people in your community respond to clinics asking about their SDOH?	
3.3.2 If clinics collected SDOH data, how should clinics use or respond to that information?	
3.3.3 Do you think the state of Minnesota should require that clinics collect this information and consider these issues in terms of financial reimbursement?	

### Analysis

We analyzed the qualitative data from the small and large group discussions using Thematic Analysis [25, 26]. Each CET Community Lead who facilitated the small group discussions used the audio-taped small group sessions to expand the original notes and ensure the notes accurately captured the discussion, while simultaneously translating from Hmong, Somali and Spanish into English as necessary. They manually coded the English language notes according to a coding tree [25] based on National Quality Forum’s 2017 Health Equity Framework [27] and then entered their coded data into one computer-based Excel spreadsheet. By reflecting on the ideas that emerged in the large and small group discussions, the full CET collectively identified the major characteristics of quality primary healthcare clinics, and then identified major themes that encompassed them. Each CET Community Lead then reviewed their coded discussions to identify which codes fit with these major characteristics and themes and then highlighted appropriate quotes. We sent the preliminary results to 17 participants who had expressed interest in reading and giving input on the results, as a participatory member-checking process. The seven participants who responded expressed agreement with the major ideas and gave additional input, which was included in the final analysis. We chose our final quotes to ensure representation from all seven communities.

## Results

The self-described characteristics of the 20 community leaders who participated in the listening sessions are in Table 2. The identified three themes about quality primary healthcare clinics and representative quotes are in Table 3.

**Table 2:** Characteristics of Community Leaders

Self-identified Characteristics	Result
Gender - N	
Women	11
Men	7
Transgender	1
Non-binary	1
Age, mean age in years (range)	45 (26-65) years
Communities - N	
African-American/ Black	3
LGBTQTS	3
Hmong	3
Latino/a/x	4
Native American	1
Somali	4
White	2
Country of Origin- N	
USA	8
Other	12
5 Somalia	
2 Colombia	
2 Laos	
1 Chile	
1 Korea	
1 Peru	
1 Turtle Island, a Native American land

**Table 3:** Themes with Participant Quotes

**Theme #1: Quality Clinics Utilize Structures and Processes that Support Health Equity**	
Recognize and address historical trauma, structural racism, and SDOH *“Clinics and clinicians should be prepared to treat the person as a “whole person”. That means that many patients have issues that make the specific disease almost non-important. For example, a patient’s dilemma is that her child is sick, but she also has another baby to take care of, her rent is not paid, her partner is “being snarky”, she does not know how to navigate the system and would take more time to learn. How can a patient find an advocate to help her through her many struggles?”* (LGBTQTS) *“If a clinic has all the resources in terms of housing, employment, or legal information—such as domestic violence, I can get help getting a restraining order—or if there is a person to refer me (and) at the same time (tell me), ‘We will care for your health, and this (resource) will relieve your pain and stress’, it’s very important to me and the Hmong community because we don’t know the language, (the) knowledge, so we don’t know where the information is.”* (Hmong) *“One of the current issues for (the) Latino community is deportation, which has ripple effects: (the) deportation of one person could affect an entire group of people, from his children who will be unable to see their father at all, to a wife who will be suddenly a single mother. ...A clinic providing (connections) for those people with resources in the community, the entire group of people could find relief: churches, low-fee attorneys, organizations helping Latinos, food pantries, school counselors, county workers*.” Latino/a/x *“We see being healthy as a right – it was written as a right into our treaties, and we see this in our (Native) teachings, but…because of trauma, I think Native communities struggle with feeling worthy of being healthy.”* (Native American) *“You know sometimes when people who just recently arrived, they have been through so much trauma. Like my grandma for example, when she came in, she said ‘I have body aches’ and later we ended up going to a therapist ... Understanding what these people went through helps so much. So, it is important for doctors to have background knowledge about what the person dealt with.”* (Somali) *“A primary care visit, to me, it’s not just I’m treating you right now, today, it’s we’re treating your overall health and we understand that’s something bothering you today that’s why you’re coming in, but let’s take an overall view of your health, whether it is diabetes, asthma, addiction. Maybe there’s some mental health challenges or some social and economic challenges that is greatly affecting this person’s overall health*.” (White)	
Have real representation by patients and community members *“In order to build trust, patients need (to) receive treatment from personnel who represent their own groups or from someone who is culturally competent in their language and culture. I heard a patient who was struggling with economic issues say, ‘The doctor is telling me I have to eat a healthy, balanced meal, with fruits and vegetables. How am I going to tell my wife that, since we hardly have enough money to get some food?’ When I asked the patient why he didn’t tell the doctor the truth, he explained that it was embarrassing enough to have to tell the non-Latino doctor, but that the Spanish interpreter also would hear it.”* Latino/a/x *“Clinic leadership should hire people who are Somali. For example, if the clinic is in neighborhood where Somalis live, they should hire interpreters from the community, but how much better (it would be) if they hired people from the community to provide services and to lead the clinic.”* (Somali)	
Report on health disparity data, goals, and efforts *“In order to make a complaint, patients have to feel empowered, feel safe and not worried that clinics or staff will retaliate. In native communities, they often don’t feel empowered enough to even complain at an official level. […]Can the clinic help people effectively complain? Because their voices collectively matter. How do we tell communities that their voices and experiences matter? This is how we can get the system to listen to them. We may have to craft our own creative ways to collect that.”* (Native American)	
Improve access to care through solutions to barriers and integrated services *“(At some clinics) I don’t think they really explain what’s going on, I mean, when you’re at the community clinic…they don’t explain anything. They just move you through, and after sitting (there for) 2-3 hours, you just want to leave…and after spending the money for the bus to get there (in time for) your appointment, and you are going to try to get back (on time), but they sit you there for 2 hours. They’ll get you checked in, but you’re sitting there for 2 hours. Now my bus pass is (expired) and I’m going to have to walk home.”* (LGBTQTS)	
Support healthcare system navigation *“To build trust, (clinics should) have (a) liaison to help patients feel comfortable, have conversation to show support for the patient, (and) have a team that collaborates so that patients don’t have to repeat themselves because it is tiresome. (…) When a family doesn’t feel empowered to be their child’s advocate, you need to encourage them to ask questions and help them be empowered to be their own advocates.”* (Native American)	
Create welcoming, private, and safe clinic environments *“Make sure that staff is trained in HIPPA. Privacy is important. Do not leave my private information for others to see. We do not trust doctors and we do not trust people with our information. Talk in a low voice instead of screaming. There are always people behind who can hear. ... Information should be private and treated with respect. This way I will come back to this clinic.”* (Black/African American) *“What is it like when you go into the clinic, is it all straight, white people?….What’s the literature in the waiting room? Are there gay magazines, are there people of color on them? And around bathrooms and bathroom management…are there single stall bathrooms available? How has the clinic chosen to label the bathrooms? How do I fit within the physicality of the clinic – do I see myself represented?”* (LGBTQTS) *“Showing respect can be as easy as asking for permission. For instance, when a doctor needs to examine a Hmong elder’s head, the doctor should ask for permission ahead of time. This act demonstrates that the doctor acknowledges and respects the patient’s control for their personal space and body. Small gestures like asking for permission contribute to building a trusting relationship between patients, doctors and staff.”* (Hmong) *“My aunt has diabetes. I went with her to see a doctor and the doctor didn’t see her as she is. She’s an elderly woman and has never been educated. He told her how to manage her diabetes, but she didn’t understand the whole scope of it or why. He told her “no more tea” and didn’t discuss it further with her. He doesn’t understand how tea is part of our culture. If he understood our culture, he could treat her well and he could reach her. Now she constantly goes to the ER instead of going to her doctor. She needs education and help to learn how she can eat.”* (Somali) *“Having a calming, healing environment (is important). I know that (my clinic) has a chaotic environment, and with anxiety, sometimes, I have trouble being in the waiting area (because) you’re in everybody’s business. I think it doesn’t really respect patients’ confidentiality and privacy.* (White)	
**Theme #2: Quality Clinics Provide Respectful, Trusting, and Effective Relationships with Patients and Communities**	
Support effective and longitudinal clinician-patient relationships *“People do not want to share a lot earlier in the relationship. The relationship must be built to have trust. Some clinics develop trust faster than others. If you trust the clinic you will be able to share more, your whole experience.”* (Black/ African American) *“For those with drug and alcohol problem, if you don’t have a supportive environment, it’s easy to slip back into using again. Creating the supports for long-term health is really important. Like mental health, one thing is to have a regular schedule with a provider. If you can only get it once a month, it’s hard to follow up with the positive that you’re getting from it. Sometimes that relationship ends when you decide you can only go so far with a therapist or the therapist thinks they need to end the working relationship.* (White)	
Provide training for staff to improve cultural-responsiveness and be attuned to unconscious bias *“Respect for elders, how the staff in the clinic acknowledge you, instead of calling you by your first name….I know that this is not the culture, but treating a client as Mr., and Mrs. first is respectful and you may give permission to call you by your first name.”* (Black/ African American) *“For me, when we go to the clinic, we are scared and when we get there, we are constantly thinking that they (staff/doctors) might not know our language, maybe they’ll have an interpreter. ‘Is the interpreter going to interpret correctly or not? Will they understand me?’ When people need interpreters, then they need more time, not only to interpret, but there is also a cultural piece. Hmong are relationship people--we need more time to build relationships with providers and explain things fully so we don’t get embarrassed.”* (Hmong) *“Cultural training must be available to the clinic staff at all levels. Clinics can even invite patients to talk about ways to improve services so the services fit with patients’ culture desires and needs. My aunt has diabetes. I went with her to see a doctor and the doctor didn’t see her as she is. She is an elderly woman and has never been educated. He told her how to manage her diabetes but she didn’t understand the whole scope of it or why. He told her ‘no more tea’ and didn’t discuss it further with her. He doesn’t understand how tea is a part of our culture. If he understood our culture, he could treat her well, and he could reach her. Now she constantly goes to the ER instead of going to her doctor. She needs education and help to learn how she can eat*.” (Somali)	
Provide culturally-relevant patient education *“Adopting healthy lifestyles is key to addressing many health issues in our community. However, there are many issues that block this useful tool to reaching many people in the community. From lack of healthcare access due to education and financial constraints to health system design, communities use the health system when they are sick or as a ‘last resort’ rather than as a tool to stay healthy and prevent diseases. … They need culturally and linguistically appropriate education and information about health promotion, so people can understand and can institute healthy lifestyles.”* Latino/a/x	
Integrate family and community-based strategies for health promotion and education *“Family members could provide important information about the patient. Latino patients are used to going to their doctor’s appointment with their spouse, children, in-laws, godmother. Often times, these family members could provide information that is useful for the provider, to give better service to the patient.”* Latino/a/x	
**Theme #3: Funding Based on Current Quality Measures Perpetuates Health Inequities**	
*“Regarding diabetes treatment, if patients continue to smoke, (then) the clinic automatically fails, (and) doesn’t get their funding…but that (smoking) is a trauma thing, you know?”* (LGBTQTS) *“The current system for reimbursement is not the best practice for communities like ours. I don’t think that a clinic (in suburbia with better quality scores) is doing any better job than a clinic in Minneapolis that is dealing with other factors. We know that only 10% of clinical factors contribute to health, and the rest is related to social factors, so social factors are a bigger component of dealing with health problems and getting the results that would rank a clinic or provider higher or lower on the current scale.”* (Somali) *“I agree, I don’t think it (payment) should be tied to it (services), because the community you are serving should be the community you are focused on, and you cannot talk about the Somali community without addressing housing issue, food issue, income issue, transportation issue. There are people that can’t even come to seek services because they are dealing with those. I think this whole rating system is deeply flawed and it seems like it benefits white people more than it benefits people of color and I think maybe the whole thing should just be scrapped and a new system should be approached based on our input. What I’m hearing is that the people most in need, those in clinic serving them could be reimbursed at a lower rate than ...other people and to me. that sounds really bad. It (financial remuneration) should be based on the people they are serving--what services do you provide and how are the people you are serving receiving the services you provide?”* (Somali)	

### Theme #1: Quality clinics utilize structures and processes that support healthcare equity

#### Recognize and address historical trauma, structural racism, and social determinants of health (SDOH)

Many community leaders discussed how the social injustices resulting from historical trauma, institutional racism, and structural inequities have negatively impacted the health of their communities. These mechanisms have contributed to communities’ high disease burdens, difficulties accessing healthcare, and a lack of trust in the healthcare system. Participants indicated that these complex and interconnected mechanisms cause physiological and psychological stress from repeated daily inequities, which contribute to chronic diseases.

Recognizing and addressing historical trauma, structural racism, and SDOH in these communities is an important contributor to healing for patients. Primary healthcare clinics need to improve their ability to identify, understand, and address social factors that influence health, as well as adjust clinics’ healthcare processes so they do not perpetuate inequities.

*“Clinicians should be aware of types of trauma in our community and understand what trauma is. Clinician[s] should be trained on how trauma looks like. Sometimes the patient herself/himself does not know she/he has been traumatized. The clinic visit could be a trigger point to realize about a trauma if the clinician is trained properly.”* (Black/ African American)

Providers should be trained in how to appropriately inquire about historical trauma, structural racism, and SDOH as this helps alleviate patients’ fears. They should consider these issues in both diagnosis and in treatment, making decisions in collaboration with patients. Clinics should have structures that include “real” representation from the clinic disparities. In addition, clinics should authentically engage and partner with community organizations to address the societal issues that negatively influence health.

#### Have real representation by patients and community members

Community members of the patient populations that are served at each clinic need to be represented throughout the clinic including staff, clinicians, and clinic managers, to executive leaders and board members. “Real” representation means they have authority and play active roles in the decision-making process, in contrast to “token” representation when people do not have meaningful input or decision-making power. The current power structure means that healthcare systems tell communities “you should be this”, when communities instead should more actively define their health and healthcare by being immersed in the organizational structure. Clinics need to intentionally cultivate relationships with trusted community members and leaders to best develop effective partnerships, which can contribute to improved patient trust, communication, connection, and healing relationships.

*“For me here in Minnesota, I mean, we have our (sexual health) programs, they’re mostly run by white women from suburbia, and we’re talking about sexual, minority health, and these are middle class white women who have no idea, and take this framework that sex should be monogamous for a lifetime, and yet we don’t live in that world… And then, when people from our communities are working in those clinics, they are just there, they do not have real power. We do not have real representation. What we need is real representation.”* (LGBTQTS)

#### Report on health disparity data, goals, and efforts

Clinics should report on their health disparity efforts by creating an “Equity Dashboard” that highlights existing health disparities and illustrates directions for progress and improvement. Collecting and displaying data can lead to improved understanding of current practices, goal setting, and accountability.

*“How do systems quantify feelings of discrimination? How do they collect that? Systems want to know what the racist action that their personnel said or did. Do they collect discrimination complaints? Do they know how many families feel discriminated against, based on their care? Are they collecting this data and if not, why not? How will they know if they have a problem? If they do not collect the data, maybe they are saying they don’t want to know.”* (Native American)

The dashboard could include: a) clinic policy leadership level data; b) clinic process data; c) clinic outcome data; and d) patients’ negative experiences with clinic processes and clinic relationships, such as being treated with disrespect/discrimination or stereotyping. An “Equity Dashboard” could help clinics explore and display how SDOH affect families and communities, how structural racism impacts community health, and how institutions respond to concerns of inequities and discrimination from patients.

In discussing whether or not clinics should be required to collect SDOH information, opinions were mixed. Participants saw potential value in providers being able to better understand patients in the context of their lives and refer patients to appropriate community programs and agencies. Potential harms included patients’ confidentiality and desire to keep this information private, increased vulnerability, and potential for discrimination. Community leaders thought perhaps it would be best for communities to be able to collect and manage their own data, being able to set their own priorities. Ultimately, they thought it was best for clinicians to respectfully ask patients about their social situations and clinics could collect the data anonymously.

#### Improve access to care through solutions to barriers and integrated services

Improving patients’ access to care includes aspects outside of the clinic (insurance, transportation, location, etc.) and inside the clinic (hours, appointments, interpreters, etc.). Increasing the prevalence of integrated services, like “one-stop shops” for healthcare that includes physical and mental health, will improve patient and community health through ease of access.

*“For many patients, transportation can be a main factor in accessing health care. There are many low-income patients who do not own a car or cannot afford car insurance, and they depend solely on public transportation. Most clinics cannot or do not accommodate late arrivals, and then deny services to patients who arrive late. Not receiving service blocks these patients from needed care, as well as produces a sense of rejection, impotence and discouragement as their time and financial efforts are wasted.”* Latino/a/x

Traditionally, healthcare clinics have focused on physical health, and have relegated mental health to special mental health services. Patients, families, and communities could benefit from the expansion of mental health services to be diffused throughout the primary healthcare system. Clinics should also partner with community spiritual, social, and mental health healers.

Although not directly responsible for the financial burden of care, clinics could support long- and short-term solutions for the high cost of care. Long-term solutions should include universal healthcare coverage, while short-term solutions could include: clinic-based discount programs; providers prescribing medications that patients can afford or are covered by insurance; and pharmacists connect with pharmaceutical company assistance programs.

#### Support healthcare system navigation

In order to best navigate the clinic and healthcare system, patients need to understand how the system works. Transparent clinic processes will help patients understand how the system can help meet their needs (i.e., understanding diagnoses, treatment plans, test results, medical/community referrals, and follow-up care). Effective communication then must make these transparent processes easy to understand by considering patients’ language, health literacy, numeracy, and technology skills.

*“[Clinics should] not leave it to the individual directly and (they should be) more involved than just say ‘This is what you have and I expect you to manage it’….[Clinics should] have a guideline or help….to train someone on how to manage (their) care, or train family members to help them manage their care….don’t expect them to figure out how to manage their own care when it’s complicated enough that someone who is born in the state can’t even manage it….”* (Somali)

#### Create welcoming, private, and safe clinic environments

A welcoming environment requires clinics to have a high standard of professionalism that reflects the needs of the communities they serve. Healing environments reduce stressors associated with the effects of societal discrimination and disrespect. Clinic spaces could include space and activities for children, artwork from local artists, healing gardens, and quiet spaces for reflection and prayer. Furthermore, providing confidential care in a private environment is respectful of patients and leads to a trusting relationship between patients and clinic staff.

*“It is important how you are treated at the front desk. When you are sick and you see the unhappy faces of the front desk staff, then it makes you twice as sick. Sometimes when you check in, they don’t even look up at you and it makes you angry and you don’t want to be there. The relationship between the mind and body is important. If you don’t value that, then you can’t contribute to the improvement of the patient’s health.” (Hmong)*

### Theme #2: Quality clinics offer effective relationships, education, and health promotion

#### Support effective and longitudinal clinician-patient relationships

Patients want long-term personal relationships with their primary care clinicians. This relationship should include respect for patients as individuals, acknowledgement and not dismissal of patient concerns, and respect for patients’ life choices. Patients desire support and advice for all of their healthcare-related needs, including acute and chronic conditions, preventative care, healthy lifestyle, and overall wellness.

*“Patients prefer to see the same provider regularly so as to form a trusting relationship. But when the scheduling staff does not or cannot schedule appointments with [the] same provider, then the patient usually does not share information that may be relevant to the reason for the visit.”* Latino/a/x

Clinics should provide adequate resources and time to help patients develop effective and therapeutic relationships with providers. Clinicians need adequate time to establish personal relationships, provide culturally-responsive care, and identify and respond to relevant SDOH factors. Trained medical interpreters are also a necessity to best serve non-English speaking and low English proficiency patients.

#### Provide training for staff to improve cultural-responsiveness and be attuned to unconscious bias

In order to help patients feel valued and respected, all clinic staff should be required to complete cultural-responsiveness training. Such training aims to mitigate implicit biases, stereotyping, and discrimination.

*“If you’re competent, then your forms and everything else will reflect that you have an understanding of who the people are that are coming in to use your services.”* (LGBTQTS)

Providers and clinic staff who understand a group’s history, culture, and healthcare practices (i.e., herbal medicine, massage, or prayer) can provide more patient-centered care and help avoid patients feeling vulnerable, misunderstood and stereotyped. Staff need to provide excellent customer service without making assumptions, reinforcing stereotypes, or passing judgment based on how people look, dress, or speak. Overall, culturally-responsive care improves patient trust, shared decision-making, patient engagement, and follow-through with healthcare goals and plans.

#### Provide culturally-relevant patient education

Traditionally, clinics have focused on doctor-dominated disease diagnosis and treatment with patients being dependent on clinicians. The shift towards focusing on prevention, health promotion, and patient empowerment for healthy goals and independence for chronic disease-self-management needs to continue.

*“It would help if the clinic held monthly seminars for the community. If you teach twenty people, they are connected with hundreds of other different people, as people are inter-connected and related in the Somali community. The person leading the sessions should be someone from the community, otherwise people attending may say, ‘Wait a minute- this is another “cadaan” (white) person telling me what I should be doing with my life’, and then not listen.”* (Somali)

Effective patient education is holistic and tailored to individuals in the context of their family and community. It must remain consistent with patients’ preferred language, literacy, and learning styles, while also considering peoples’ cultural values of health and healing, and respecting patient’s intersectional identities.

#### Integrate family and community-based strategies for health promotion

Individually-focused care can fracture the family and community structures, isolating individuals from their support network. Family-focused health promotion and education can support healthy lifestyles for the whole family, while using patients’ support systems to empower them. Culturally-responsive healthcare for communities that value a collaborative versus individualistic view of health may mean including family and friends during patient visits.

*“When we talk about diet and changes, we need to consider the household. We are family-oriented so to eat healthier, exercise, can’t do that unless we change entire family lifestyles. We have to keep everyone accountable, to ask who lives in the household and asking others in the home and how to change the family structure and community to make every one healthy.”* (Hmong)

### Theme #3: Funding based on current quality measures perpetuates health inequities

Community leaders generally disparaged the reality of the current system, where privileged patients and communities have higher quality scores than impoverished and marginalized clinic populations. They recognized that if the clinics serving privileged communities receive increased reimbursement while clinics serving impoverished communities receive less money (i.e. structural disparities), the social injustice of our healthcare system and its disparities will continue. They emphasized the importance of how social and structural determinants of health disproportionately impact the funds and resources for their communities, and how the current system perpetuates the inequities in their communities. They criticized policymakers and decision-makers who set budgets and priorities that do not align with low-income communities of color and communities that experience the greatest health disparities. Some participants advocated for stopping the inequitable process altogether, while others made suggestions to improve the system (i.e., create a system that modifies payment by community’s socio-economic factors; reimburse based on community’s social needs; reimburse based on time expended to meet patients’ needs). Other participants shared ideas of what the funding priorities should be (i.e., health education about healthy lifestyles to prevent chronic diseases; community services outside of clinic processes) and how the funds should be spent to improve patient experience (i.e., hiring staff from the community; training staff to be culturally sensitive).

*“The state will prioritize the taxpayer that pays the most and the one who screams the loudest—they are not the poor and not people of color, which is what creates great disparities.”* (LGBTQTS)

*“The current system for reimbursement is not the best practice for communities like ours. I don’t think that a clinic (in suburbia) is doing any better job than a clinic in Minneapolis that is dealing with other factors. We know that only 10% of clinical factors contribute to health, and the rest is related to social factors, so social factors are a bigger component of dealing with health problems and getting the results that would rank a clinic or provider higher or lower on the current scale.”* (Somali)

## Discussion

Twenty community leaders from seven urban communities (Black/African American, LGBTQTS), Hmong, Latino/a/x, Native American, Somali, and White) in Minneapolis-Saint Paul, Minnesota, USA, participated in listening sessions as key informants to share their perspectives about what their communities wanted in quality primary healthcare. Their responses are organized by three themes: #1: Quality Clinics Utilize Structures and Processes that Support Healthcare Equity; #2: Quality Clinics Offer Culturally Appropriate Relationships, Education, and Health Promotion; and #3: Funding Based on Current Quality Measures Perpetuates Health Inequities.

### Evidence-based discussion of participants’ quality recommendations

These community leaders’ perspectives about quality primary care and their recommendations about how to achieve it are supported by existing literature. Their assertions that health is influenced by social risk factors, historical trauma, and structural racism (Theme #1) has been demonstrated by many studies that illustrate how social and economic factors are major determinants of health and well-being [28, 29]. Similarly, the impact of historical trauma and structural racism adversely affect mental health as well as physical health [30]. Pathways between racism and health outcomes have been increasingly evaluated, including economic injustice and social deprivation, environmental and occupational health inequities, psychosocial trauma, targeted marketing of health-harming products, inadequate healthcare, and maladaptive coping behaviors [30, 31]. Focusing on structural racism as a key determinant of population health is essential to advancing health equity [32].

There is moderate evidence for our community leaders’ practical recommendations for responding to social risk factors, historical trauma, and structural racism. They proposed that quality primary care clinics need to have equitable leadership structures, employ staff who both belong to and are respected in the communities, and include community members in clinic systems with decision-making power. Providers and health systems increasingly recognize the value of including patient and community perspectives in healthcare systems, particularly for quality improvement and research endeavors [33]. Integrating patients with direct personal experience in health systems can provide insights and raise concerns that may not be noted by health professionals alone [34] and promote patient-centered practice improvements [35]. Systematic reviews of patient, family, and community advisory boards have indicated that such groups best contribute to patient-facing services that may improve patient-centeredness and satisfaction but are difficult to evaluate in terms of impact on health outcomes. Therefore, more prospective clinical outcome data is needed [36] to monitor process and progress on health disparities. Although some states, including Minnesota, already issue an annual Health Equity of Care Report [22], individual clinics and their patients may benefit from displaying and tracking health disparities through an instrument such as an “Equity Dashboard.” [27].

The community leaders’ list of items that constitute quality primary care in Theme #2 have in common a call for more deliberate culturally specific processes that are responsive to the needs of patients and the communities that the clinics serve. Among the recommendations, there is evidence for improvements in patient satisfaction and understanding among several ethnic and religious groups across multiple health conditions when effective, culturally-appropriate education and resources are provided [37, 38]. Although there is little research about the effects of cultural responsiveness training interventions on healthcare disparities [39], a 2014 Cochrane review indicated mild improvements in patient, provider, and healthcare organization outcomes with no studies showing adverse outcomes [40]. A recent article makes a powerful case for cultural safety approaches as distinct from cultural sensitivity/competency as being more effective in addressing health inequities [41]. Perhaps the strongest evidence-based recommendation from our community leaders was to support long-term relationships with primary care providers (Theme #2). Longitudinal continuous relationships between primary care providers and patients are related to the overall success of primary care [42], patient satisfaction [43], reduced healthcare costs and outcomes [44], including lower death rates [45]. This may be especially important to communities with a warranted distrust of the healthcare system.

The community leaders’ recommendations for quality clinics in Themes #1 and #2 reflect the tenets of patient-centered medical homes (PCMHs) such as integrated mental health services, enhanced access, and care coordination to help navigate the healthcare system. The PCMH has been touted as a way to promote health equity [46] and has been shown to improve some patient outcomes [47], reduce some health disparities [48], and increase patient satisfaction [49]. Specifically, same-day appointments have been shown to increase patient satisfaction, decrease emergency department usage, and improve cost-effectiveness of care [50]. Moreover, improvements to interpreter services in the outpatient settings can produce more efficacious and efficient patient care, as well as reduce malpractice claims [51]. Additional legal and social services have demonstrated reductions in overall system costs [52, 53].

The third theme arose from our asking community leaders’ about tying clinic and provider reimbursement to current quality measures. Their response was clear: this financial arrangement would contribute to increased health disparities, particularly if conducted without adjusting for social risk factors. The community leaders’ concerns are supported by strong and rapidly growing evidence. Providers serving a higher proportion of disadvantaged patients have been shown to have worse quality scores [10] as current quality metrics do not typically consider structural and social factors that contribute to health and quality scores [15]. Tying quality metrics to reimbursement strategies produce unintended consequences [13] such as reducing access and increasing healthcare disparities in disadvantaged populations [54] and inappropriately labeling clinics and providers as poor performers [23]. Given the disadvantages placed on providers serving populations most affected by health disparities, it is already difficult to recruit PCPs and specialists to predominantly minority neighborhoods [55]. Adjusting provider pay and clinic resources based on inequitable measures may only exacerbate this [7].

### Theoretical grounding and contribution to current literature

Leading experts at the intersection of primary care quality measurement and health equity such as Starfield [18] and Stange/Etz [19], have described the complicated relationship between quality and equity. Both researchers acknowledge health equity as a necessary component of quality primary care, and Starfield demonstrated multiple pathways from quality *measurement* to health *inequity*. Starfield concludes that “Efforts to improve average health, i.e., population-wide rates of morbidity and mortality, are generally associated with increasing inequities, because new and effective interventions often reach the more advantaged first. Also, influences with high relative risk of poor health are not necessarily appropriate targets for equity-focused interventions, as their frequency may be low and hence not contribute much to reductions in inequity overall [56]. In the context of our study, the bottleneck to improving the health of marginalized populations can vary according to their SDOH and community specific needs. Moreover, rewarding clinicians to pursue quality metrics most relevant to the upper-middle class seems to systematically discriminate against marginalized populations [7, 15].

One way to block the pathway from improving quality metrics to increasing inequity described by Starfield is instituting the Person-Centered Primary Care Measure (PCPCM), recently proposed by Etz et al [57]. The PCPCM is a comprehensive measure that requires clinicians to center their quality efforts on the needs of the individual patient, as a balancing measure to population-centered metrics that may miss the mark for that patient. A second way to block the quality metrics-inequity pathway is to conceive of health equity as an explicit element of quality primary care. Stange and Etz cite collaborative leadership and stakeholder participation as principles that can be measured, and can be included in healthcare quality and equity metrics [19]. Our study suggests the potential for greater health equity by incorporating the voices of marginalized community leaders in primary care quality metric development.

Moreover, our study points to the potential for improving health outcomes and health equity through the addition of community-specific metrics. Such meso-level metrics may be an important contribution between the traditional macro-level metrics of entire populations and new PCPCM micro-level metrics for individual patients. Exploration of community-level metrics is an exciting prospect and we recommend further conceptual and empirical research.

### Limitations

The participants were limited to a small number of people from each community and each community had a different number of people represented. While the quotes give a flavor of each communities’ perspectives, the low number of participants per community required our analyzing the data as a whole report, and precluded our sub-analyzing the data by each community. Indeed, characterizing different community perspectives would require a more substantial qualitative and a subsequent quantitative investigation. Overall, this study could be seen as an initial investigation of “key informants” who shared their perspectives about their communities’ experiences before conducting a more in-depth evaluation of community members’ perspectives about quality care and quality metrics. Nonetheless, these themes have been supported by prior research.

## Conclusion

As we approach more than 1 year of the COVID-19 pandemic, we believe that the time for action to redress societal inequity in health care is now. Our study’s diverse community leaders identified ideal characteristics of primary healthcare that could address health inequities and promote quality care. They expressed concerns that linking clinic payment with quality metrics without considering SDOH perpetuates current social injustices in the healthcare system. Their insights and recommendations can guide us to a system that more equitably measures and resources primary care clinics for quality primary healthcare.

## Data Availability

The de-identified interview transcripts and spreadsheets of codes and quotes created for analysis are available from the corresponding author on reasonable request.

## Abbreviations

CBPR: Community-based Participatory Research
CET: Community Engagement Team
LGBTQTS: Lesbian-Gay-Bisexual-Transgender-Queer-Two Spirit
MDH: Minnesota Department of Health
NQF: National Quality Forum
PCP: Primary care provider
QMEP: Quality Measurement Enhancement Project
SDOH: Social-structural determinants of health
SQRMS: Statewide Quality Reporting and Measurement

## Initials of authors

KACP: Kathleen A Culhane-Pera, MD MA
SLP: Shannon L Pergament, MSW MPH
MYK: Maiyia Y Kasouaher, PhD
AMP: Andrew M Pattock, MD
ND: Naima Dhore, BA
CNK: Cindy N Kaigama, MA
MA: Marcela Alison, MBA ICP
MS: Michael Scandrett, JD
MST: Mai See Thao, PhD
DJS: David J Satin, MD
