# Correction to: Diverse community leaders’ perspectives about quality primary healthcare and healthcare measurement: qualitative community-based participatory research

**DOI:** 10.1186/s12939-021-01586-0

**Published:** 2021-12-27

**Authors:** Kathleen A. Culhane-Pera, Shannon L. Pergament, Maiyia Y. Kasouaher, Andrew M. Pattock, Naima Dhore, Cindy N. Kaigama, Marcela Alison, Michael Scandrett, Mai See Thao, David J. Satin

**Affiliations:** 1 SoLaHmo Partnership for Health and Wellness, Minnesota Community Care, Inc., 895 E 7th, MN 55106 St. Paul, USA; 2 Program in Health Disparities Research, University of Minnesota, 717 Delaware St. SE, Minneapolis, MN 55414 USA; 3 Department of Family and Community Medicine, University of Minnesota, 420 Delaware Street SE, Minneapolis, MN 55455 USA; 4 Minnesota Health Care Safety Net Coalition, 1113 East Franklin Ave #202B, Minneapolis, MN 55404 USA; 5 Department of Anthropology, Global Religions and Cultures, University of Wisconsin-Oshkosh, 800 Algoma Blvd, Oshkosh, WI 54901 USA

**Correction to: Int J Equity Health 20, 226 (2021)**

**https://doi.org/10.1186/s12939-021-01558-4**

After publication of this article [1], the authors reported that various author (text) corrections have been carried out in the web version, but not in the pdf, e.g.,

- ▪ Table 2 does not have appropriate indentations;
- ▪ Table 3 is hard to read due to poor formatting;
- ▪ in Table 2 and elsewhere, GLBTTQ should be LGBTQTS and Latino is to be Latino/a/x;
- ▪ in Table 2: African-American/Black should be Black/African American;
- ▪ and some minor text corrections.

The original article [1] has been updated.
